# Iodine and doxorubicin, a good combination for mammary cancer treatment: antineoplastic adjuvancy, chemoresistance inhibition, and cardioprotection

**DOI:** 10.1186/1476-4598-12-45

**Published:** 2013-05-24

**Authors:** Yunuen Alfaro, Guadalupe Delgado, Alfonso Cárabez, Brenda Anguiano, Carmen Aceves

**Affiliations:** 1 Instituto de Neurobiología, Universidad Nacional Autónoma de México, Campus-Juriquilla, Querétaro 76230, México

**Keywords:** Mammary cancer, MNU, Iodine, Doxorubicin, Chemoresistance, PPARγ, Cardioprotection

## Abstract

**Background:**

Although mammary cancer (MC) is the most common malignant neoplasia in women, the mortality for this cancer has decreased principally because of early detection and the use of neoadjuvant chemotherapy. Of several preparations that cause MC regression, doxorubicin (DOX) is the most active, first-line monotherapeutic. Nevertheless, its use is limited due to the rapid development of chemoresistance and to the cardiotoxicity caused by free radicals. In previous studies we have shown that supplementation with molecular iodine (I_2_) has a powerful antineoplastic effect in methylnitrosourea (MNU)-induced experimental models of MC. These studies also showed a consistent antioxidant effect of I_2_ in normal and tumoral tissues.

**Methods:**

Here, we analyzed the effect of I_2_ in combination with DOX treatment in female Sprague Dawley rats with MNU-induced MC. In the first experiment (short) animals were treated with the therapeutic DOX dose (16 mg/kg) or with lower doses (8 and 4 mg/Kg), in each case with and without 0.05% I_2_ in drinking water. Iodine treatment began on day 0, a single dose of DOX was injected (ip) on day 2, and the analysis was carried out on day 7. In the second experiment (long) animals with and without iodine supplement were treated with one or two injections of 4 mg/kg DOX (on days 0 and 14) and were analyzed on day 56.

**Results:**

At all DOX doses, the short I_2_ treatment induced adjuvant antineoplastic effects (decreased tumor size and proliferating cell nuclear antigen level) with significant protection against body weight loss and cardiotoxicity (creatine kinase MB, cardiac lipoperoxidation, and heart damage). With long-term I_2_, mammary tumor tissue became more sensitive to DOX, since a single injection of the lowest dose of DOX (4 mg/Kg) was enough to stop tumor progression and a second DOX4 injection on day 14 caused a significant and rapid decrease in tumor size, decreased the expression of chemoresistance markers (Bcl2 and survivin), and increased the expression of the apoptotic protein Bax and peroxisome proliferator-activated receptor type gamma.

**Conclusions:**

The DOX-I_2_ combination exerts antineoplastic, chemosensitivity, and cardioprotective effects and could be a promising strategy against breast cancer progression.

## Background

The two factors responsible for most breast cancer-related deaths are the ability of cancer cells to metastasize and to develop resistance to anti-cancer therapies. Indeed, resistance to chemotherapy is a major obstacle to successful treatment of breast cancer [1,2]. Anthracycline antibiotics are among the most effective and commonly used anticancer drugs, and doxorubicin (DOX) in particular is often the first choice to treat mammary cancer. Nevertheless, its use has been limited due to the rapid development of chemoresistance and cardiomyopathic side effects [1,3]. The therapeutic activity of DOX results from its intercalating into DNA, thereby inhibiting topoisomerase II and preventing DNA and RNA synthesis [4,5]. With respect to chemoresistance, it is known that DOX-resistant cells exhibit: 1) diminished Topoisomerase II expression, 2) increased production of calcium-dependent protein transglutaminases and of integrins involved in membrane stabilization, and 3) increased expression of anti-apoptotic messengers like Bcl2, Bxl, or survivin [2,6,7]. The protein survivin (Surv), the smallest member of the inhibitor of apoptosis protein (IAP) family, is expressed and required for normal fetal development but is generally absent in adult tissues. However, re-expression of Surv is observed in numerous human cancers where its presence is associated with enhanced proliferation, metastasis, and a poor prognosis [8].

Short-term adverse effects of DOX occurring within 2–3 days of administration include nausea, vomiting, neutropenia, alopecia, and arrhythmias [9]. A long-term side effect is the cardiomyophathy associated with congestive heart failure [10,11]. This chronic cardiotoxicity is likely related to iron oxidation and oxygen free radical formation rather than to antineoplastic mechanisms of the drug, since cardiomyocytes are minimally replicating cells [10]. DOX typically induces dilated cardiomyophathy with microscopic features including myofibrillar loss, mitochondrial and interstitial edema, fibroplasias, etc. Clinical serum biomarkers such as creatine kinase MB (CK-MB) or troponins may increase and serve as important, early predictors of cardiotoxicity [12,13].

Low incidences of mammary cancer and cardiovascular disease have been associated with the high iodine intake in Asian, as compared to Western populations (5280 vs 209 μg /day). Seaweeds, such as *wakame*, *nori*, or *mekabu*, are widely consumed in Asian countries and contain high quantities of iodine in several chemical forms [14,15]. In recent years several research groups including ours have demonstrated that iodine *per se* acts as an antioxidant in the whole organism and participates in the physiology and/or pathology of organs that capture iodine, such as thyroid, mammary gland, prostate, and stomach [16-19]. In mammary gland, supplementation with molecular iodine (I_2_), but not iodide (I^-^), alleviates human mastalgia and exerts potent antineoplastic and apoptotic effects on animal and human cancer. When administered over periods of weeks up to years, moderately high concentrations of I_2_ supplements (3–6 mg/day) have no deleterious effects on thyroid or general health [20-22]. Although the cellular mechanisms through which iodine exerts these effects have not been elucidated in depth, two hypotheses have been proposed: a direct action involving the antioxidant/oxidant properties of iodine, and an indirect effect through iodolipid formation. In the case of antioxidant/oxidant effects, two sets of data have been obtained showing that: a) at low or moderate concentrations, I_2_ significantly reduces lipid oxidation either by competing with reactive oxygen species (ROS) for various cellular components, or by neutralizing HO radicals, and b) at high concentrations, iodine acts as a direct oxidant, dissipating the mitochondrial membrane potential, thereby triggering mitochondrion-mediated apoptosis [18,23-25]. The indirect effect was originally postulated for thyroid tissue and involves the formation of iodolipids such as 6-iodo-5-hydroxy-8,11,14-eicosatrienoic acid (also called 6-iodolactone; 6-IL) or alpha-iodohexadecanal derived from arachidonic acid (AA) or eicosapentaenoic acid, respectively [26]. Both iodolipids exert apoptotic effects [27], and our group has demonstrated that 6-IL is present in mammary cancer cells after I_2_ supplement and is a functional ligand of peroxisome proliferator-activated receptor (PPAR) type gamma (PPARγ) [28-30]. PPARs are ligand-activated transcription factors, and three subtypes -- PPAR alpha, PPAR beta, and PPAR gamma -- have been identified [31]. Although originally described as molecular regulators of lipid metabolism, PPARγ were recently shown to play an important role in cell proliferation, differentiation, and apoptosis in many cancer cell lines including breast, prostate, and non-small-cell lung cancer [32]. Moreover, PPARγ activation potentiates the cytotoxic effect of chemotherapeutic agents such as DOX by inhibiting the expression of anti-apoptotic proteins like Bcl2 and Surv [7] or by delaying the epithelial-mesenchymal transition [33].

In the present work we evaluated the effect of I_2_ supplement in combination with DOX on the methylnitrosourea (MNU)-induced mammary cancer model. Our results show that a 7-day I_2_ supplement exerts a significant antineoplastic adjuvant effect with DOX as well as significant cardioprotection, whereas a 56-day iodine treatment is associated with enhanced DOX sensitivity, increased Bax and PPARγ, and decreased Bcl2 and Surv expression suggesting that iodine, through PPARγ expression/activation, induces differentiation and impairs the development of chemoresistance.

## Results

### Short-term DOX-I_2_ combination

**Figure 1:**
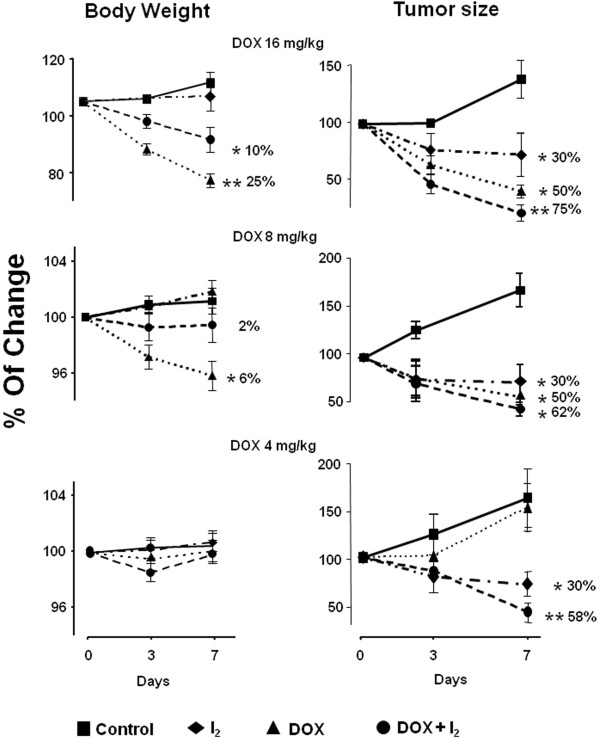
**Effect of molecular iodine(I**_**2**_**) and different doses of doxorubicin (DOX) on body weight and tumor size in MNU**-**induced rats. **Animals with tumors (2–3 cm^3^) received single injections of DOX and 0.05% I_2 _treatment (drinking water; beginning 2 days before the DOX injections). Data are expressed as the mean ± SD (n = 10). * Indicates *p* < 0.05 compared to control or **compared with DOX group.

Figure 1 summarizes body weight and tumor growth for animals given the three doses of DOX with and without I_2_ supplement. The data show that high concentrations of DOX (16 and 8 mg) were associated with dose-dependent weight loss and decreased tumor growth, but the DOX4 and I_2_ groups showed no changes in body weight gain. DOX4 alone did not decrease tumor growth, whereas the I_2_ group exhibited a 30% reduction of tumor size. When DOX and I_2_ were administered together, a significant and consistent protection against body weight loss was observed in groups treated with high doses of DOX (16 and 8 mg/Kg), and tumor size reduction was significantly greater with I_2_ than without I_2_ in the DOX16 and DOX4 groups.

**Figure 2:**
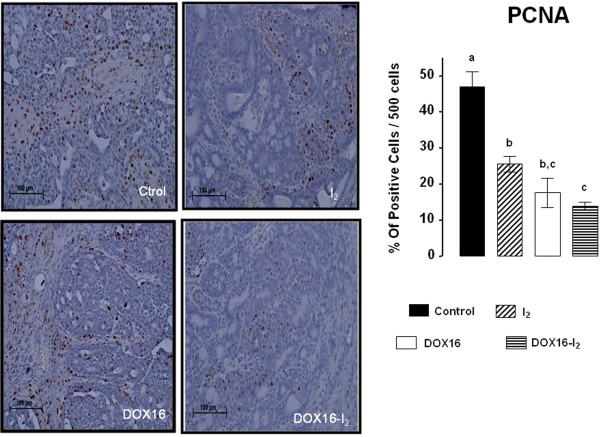
**Proliferation rate. **Immunohistochemical presence of PCNA-positive cells in tumors from control and DOX16-treated animals with and without iodine supplement for one week. PCNA-positive cells were revealed with diaminobenzidine (brown stain) and counterstained with hematoxylin (purple stain). Data are expressed as the mean ± SD (n = 6). Means with different letters indicate statistically significant differences (p < 0.05).

**Figure 3:**
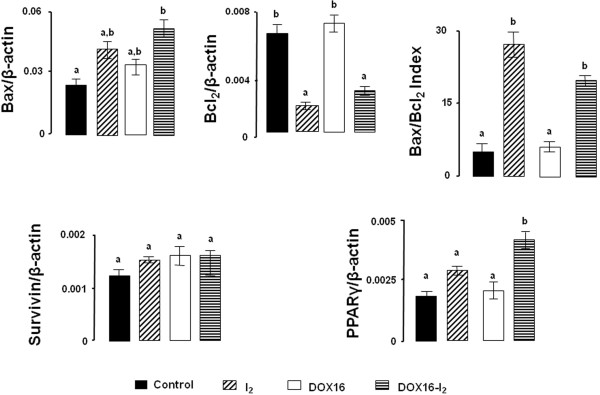
**Short**-**term effect on gene expression. **Animals with tumors (2–3 cm^3^) received a single injection of DOX16 and were treated with 0.05% I_2_ for one week. mRNA expression was measured by qPCR. β-actin mRNA was amplified to check for RNA quantity and integrity. The experiments were repeated three times with independent RNA samples. Values are expressed as mean ± SD. Means with different letters indicate statistically significant differences (p < 0.05).

Mechanisms involved in the antineoplastic effect of DOX and iodine were analyzed in animals treated with the therapeutic dose of DOX (DOX16 groups). The results showed that either DOX16 or I_2_ treatments significantly decreased the proliferation rate (measured by PCNA), its combination achieves a higher response than separately (Figure 2). Only I_2_ groups (I_2_ and DOX16-I_2_) exhibited significant increases in the apoptotic index (measured by Bax/Bcl2, Figure 3). No changes in Surv expression were observed at any dose, and only the group treated with both components (DOX16-I_2_) showed significant increases in PPARγ expression.

### Cardioprotective effect

**Figure 4:**
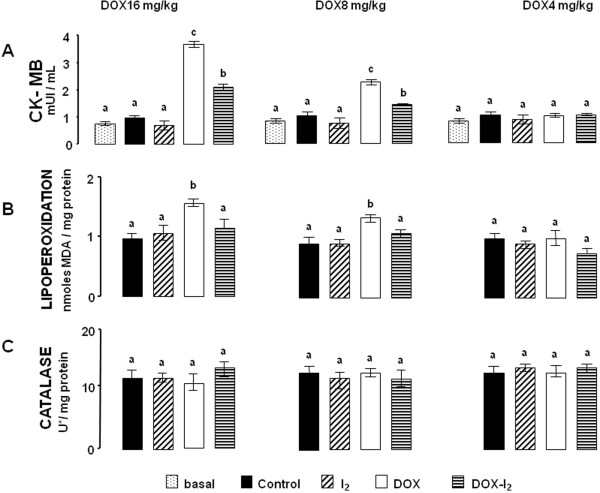
**Cardiac responses. **CK-MB serum concentration (**A**), cardiac lipoperoxidation [malondialdehyde (MDA); **B**] and catalase activity [U* = μmoles of H_2_O_2 _consumed/min; **C**] were measured in MNU-treated animals injected with saline (Control) or DOX exposed or not to 0.05% I_2 _for one week. Values are expressed as mean ± SD. Means with different letters indicate statistically significant differences (p < 0.05). Basal group in CK-MB corresponds to control animals without MNU treatment.

**Figure 5:**
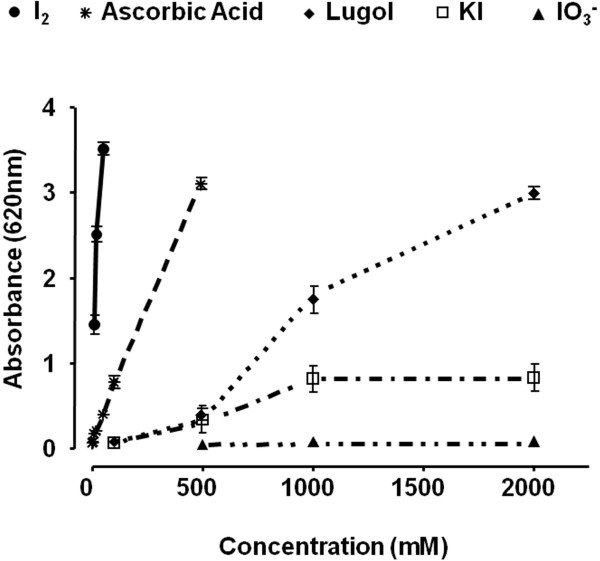
**Iodine antioxidant power. **The antioxidant capacity of different chemical forms of iodine was analyzed with the ferric reducing/antioxidant power assay (FRAP). Lugol: solution of I_2_:KI (1:3). Ascorbic acid was used as positive control. Values are expressed as mean ± SD (n = 4).

DOX16 and DOX8 treatments resulted in significant, dose-dependent increases in serum CK-MB activity, whereas DOX4 or I_2_ groups did not show any changes (Figure 4A). The co-administration of DOX and I_2_ was accompanied by a significant attenuation of the CK-MB increases generated by the high doses of DOX (DOX-16-I_2_ and DOX8-I_2_). In order to determine if I_2_ cardiac protection was related to its antioxidant effects, lipoperoxidation and catalase enzyme expression were analyzed in heart tissue. Figure 4B shows that the high levels of cardiac lipoperoxidation observed in DOX16- and DOX8-treated animals were prevented when the animals were supplemented with iodine. In contrast, catalase expression did not change, indicating no participation of this enzyme (Figure 4C). These findings agree with microscopic analysis showing significantly less heart injury in DOX16-I_2_ than in DOX16 animals, where the principal damage observed was in mitochondria, sarcomeres, and fibers (Additional file 1). Based on the *in vitro* FRAP assay, the reductive capacity of molecular iodine is 10 times higher than that of ascorbic acid, and 30 to 60 times that of other chemical forms of iodine (Figure 5).

### Long-term DOX4/I_2_ combination

**Figure 6:**
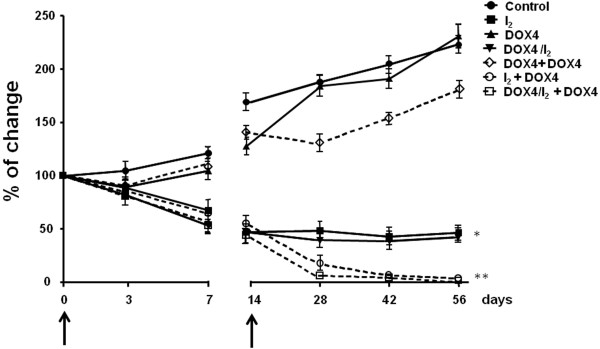
**Long**-**term effect on tumor size (% change). **Animals with tumors (2–3 cm^3^) received one (day 0) and/or two (days 0 and 14) injections of saline (Control) or DOX4 with and without I_2 _supplement, and tumor size was measured at 56 days. Single injection on day 0 of DOX4 (DOX4); I_2_ alone (I_2_); DOX4 + I_2 _(DOX4/I_2_); DOX4 + second injection (day 14) of DOX4 (DOX4 + DOX4); I_2_ with only the day 14 injection of DOX4 (I_2_ + DOX4), and DOX/I_2 _with the second injection of DOX4 on day 14 (DOX4/I_2_ + DOX4). Vertical arrows indicated DOX4 injections days. * Indicates *p* < 0.05 compared to control, **compared with I_2 _and DOX4/I_2 _groups.

**Figure 7:**
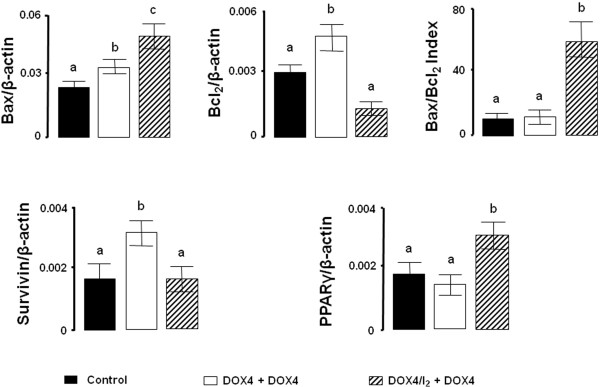
**Long-term I**_**2 **_**effect on gene expression. **Animals with tumors (2–3 cm^3^) received two injections of saline (Control) or DOX4 without (DOX4 + DOX4) or with continuous I_2 _supplement (DOX4/I_2_ + DOX4) for 56 days. mRNA expression was measured by qPCR. β-actin mRNA was amplified to check for RNA quantity and integrity. The experiments were repeated three times with independent RNA samples. Values are expressed as mean ± SD. Means with different letters are statistically significantly (p < 0.05).

In order to analyze the efficacy of long-term I_2_ supplement (56 days) as a means of preventing the chemoresistance to DOX, we designed a second series of experiments using one or two injections of the lower dose of DOX (DOX4). Figure 6 shows that DOX4 alone exerts a modest and transitory antineoplastic effect after both the first and the second injections, with tumor growth resuming a few days later. In the case of I_2_ supplement, alone or with one initial DOX4 injection (I_2_ or DOX4/I_2_), a significant and rapid decrease is observed in the tumor growth (first 7 days), but tumor size remains constant after 14 days. In contrast, the injection of one DOX4 dose after 14 days of I_2_ supplement (I_2_ + DOX4) or a second injection of DOX4 in the DOX4/I_2_ group exerts a significant adjuvant antineoplastic effect that almost eliminates the tumor mass after 42 days in both groups (Figure 6). Comparison of the groups DOX4 + DOX4 vs. DOX4/I_2_ + DOX4 shows that repeated, low concentrations of DOX induce increases in the expression of both apoptotic (Bax) and anti-apoptotic messengers (Bcl_2_ and survivin), suggesting the development of chemoresistance (Figure 7). In contrast, iodine supplement blocks the induction of anti-apoptotic messengers and favors the induction of positive apoptotic signals (Bax and PPARγ expression). The significant increase in the Bax/Bcl2 index in DOX4/I_2_ + DOX4 suggests an enhanced DOX sensitivity that correlates with the marked reduction of tumor mass observed in this group.

## Discussion

Mammary cancer is the most common malignant neoplasia in women worldwide and although many different types of cytotoxic drugs have been developed for its treatment, all are accompanied by adverse effects and in many cases by the development of drug resistance [1]. Thus, there is increasing interest in natural products that can complement conventional medicine. The present study was designed to evaluate the antineoplastic and cardioprotective effects of iodine in conjunction with DOX, the most effective and commonly used chemotherapeutic drug to treat mammary cancer [34]. Our data corroborated previously reported antineoplastic and cardiotoxic effects of DOX [13] and showed that iodine *per se* exhibited a significant antineoplastic effect in MNU-induced mammary cancer [22,29]. We also found that I_2_ potentiated the cytotoxic effect of DOX, inhibited the development of chemoresistance to it, and provided significant cardioprotection.

To understand the mechanisms involved in DOX-I_2_ action, we utilized a variety of molecular techniques to label and measure selected markers. In the present study, we demonstrate that either DOX or I_2_ alone was able to reduce proliferation by about 50%, but there was no potentiation when the two treatments were administered together. This finding suggests that cellular arrest could not be the prime mechanism involved in the adjuvant effect of I_2_. In contrast, when the apoptotic index (Bax/Bcl_2_) was analyzed, iodine had a significant adjuvant effect at both high and low DOX doses. Moreover, the finding that PPARγ expression increased only in I_2_-supplemented animals suggests that DOX potentiates the PPARγ overpexpression mediated by I_2_. It is well documented that DOX impairs the synthesis or replication of DNA, resulting in the apoptotic activation of the Bax-caspase pathway [12] and increased expression of anti-apoptotic messengers like Bcl_2_ and Surv [8], suggesting a complex interaction between apoptotic and anti-apoptotic signaling. Indeed, some authors have proposed that the long-term imbalance in this interaction could explain the development of DOX resistance that occurs when the anti-apoptosis signaling prevails [7]. Molecular iodine administration is accompanied by specific increases in PPARγ and Bax, as well as significant decreases or no changes in Bcl_2_ and Surv expression. These pro-apoptotic patterns of iodine action have been described previously in both *in vitro* and *in vivo* models and agree with the well-documented regulation of the Bax/Bcl2-caspase family by PPARγ [19,32]. Although a specific PPAR response element has not been identified for these genes, activation of PPARγ provokes a significant decrease in the expression of Blc2 and Surv in mammary tumor cell lines MCF-7 and MDA-MB231 [7]. The direct participation of PPARγ was confirmed using the specific antagonist GW9662 and by the lack of inhibition observed in cells transfected with a PPARγ dominant negative plasmid. The wide spectrum of effects exerted by PPARγ has been explained by its multiple activation pathways and transcriptional regulation of target genes: 1) classical activation to form a heterodimer with the retinoid X receptor (RXR) that binds to specific recognition sites, named the peroxisome proliferator response elements (PPRE), located within the promoter regions of PPAR-responsive genes (ligand-dependent transactivation); 2) in the absence of ligand, PPARγ and RXR could also be associated with transcriptional co-repressor complexes such as nuclear receptor co-repressor (N-CoR) or SMRT (silencing mediator of retinoid and thyroid receptors), which have deacetylation activity (HDAC) and mediate transcription repression (ligand-independent repression), or 3) the ligand-PPARγ complex could repress transcription by inhibiting the activities of other transcription factors, such as members of the NFκB and AP-1 families (ligand-dependent transrepression) [31]. It was proposed that this last repression pathway might down-regulate the expression of Bcl2 and Surv [7].

Several studies suggest that the cardiotoxicity induced by DOX is mediated by reactive oxygen species (ROS) [9]. Two different mechanisms have been identified. The first implicates semiquinone-type free radicals produced in the NADPH-dependent reductase pathway. The second mechanism includes a non-enzymatic reaction of DOX with iron that generates H_2_O_2_; the peroxide, in turn, forms hydroxyl radicals that cause oxidative injury in cellular systems [10]. In one study, the activities of three enzymes capable of detoxifying activated oxygen were determined in both heart and liver; although glutathione peroxidase activity was similar in both tissues, cardiac muscle contained 150 times less catalase and nearly four times less superoxide dismutase than liver. These results showing the low antioxidant resources of the heart, together with its very active metabolism, led the authors to suggest that the heart is particularly vulnerable to damage by free radicals generated in the presence of DOX [35,36]. Our present results corroborate the DOX cardiotoxicity: the highest values for CK-MB and LPO activity were in the DOX16 and DOX8 groups. DOX cardiotoxicity was attenuated, however, in the corresponding groups supplemented with I_2_. The specific mechanism involved in the cardioprotective effect of I_2_ is not known; indeed, this is the first report that iodine could have this effect. It is well known that other organs besides the thyroid gland are capable of taking up iodine, although heart has not been included in the list [17]. However, in a recent article [37] the authors report that myocardiocytes can produce thyroid hormone under ischemia-like conditions. They have shown that thyroglobulin, DUOX1, DUOX2, the sodium-iodide symporter, pendrin, thyroid peroxidase, and the thyroid-stimulating-hormone receptor are transiently up-regulated during this injury, suggesting that this allows cardiomyocytes to initiate cell-protective mechanisms even before local circulation is re-established. Whether a similar mechanism is triggered in these cells during DOX injury has not been explored; however, the high reductive capacity of I_2_ (FRAP assay) and its preventive effect against heart lipoperoxidation observed in the DOX16-I_2_ group suggest that iodine might be acting by a direct antioxidant mechanism, neutralizing the free radicals and preventing them from damaging other biomolecules. Indeed, this powerful antioxidant effect has been previously described as a systemic effect on whole organism (17), and is corroborated in the present study by the significant protection against body weight loss observed when animals with high Dox doses (8 and 16 mg) were treated with I_2_.

Another main finding of our work was the long-term effect of I_2_ in animals that received the lowest dose of DOX (DOX4) once or at two different times. We observed that DOX4 alone (one or two injections) could impede tumor growth only in a modest and transitory manner and that the tumoral “escape” was accompanied by increases in anti-apoptotic markers like Bcl2 and Surv. In contrast, the long-term I_2_ supplement (at least 14 days) with one or two DOX4 doses significantly enhanced the sensitivity to DOX, decreasing tumor size and blocking the increase of anti-apoptotic markers, suggesting that the pathways to chemoresistance were blocked. Moreover, the significant increases in PPARγ expression in this group, led us to propose that I_2_ affects the expression and activation of these receptors. The participation of PPARγ in chemoresistance has been extensively documented: PPARγ activation potentiated the cytotoxic effect of chemotherapeutic agents such as DOX by 1) inhibiting the expression of anti-apoptotic proteins like Bcl2 and Surv [7], and 2) inducing differentiation, decreasing cell proliferation, and increasing E-cadherin and beta-catenin expression, indicating the possible interference of PPARγ with processes like the epithelial-mesenchymal transition implicated in acquisition of the invasive phenotype [33,38,39].

## Conclusions

A robust body of evidence supports the notion that moderately high concentrations of molecular iodine exert apoptotic effects in several cancer cells as well as general antioxidant actions in the organism. In the present work we demonstrated that, through activation of PPARγ, long-term I_2_ treatment increases the tumor sensitivity to DOX, inhibits chemoresistance, and exerts cardioprotective effects, allowing a four-fold reduction in the therapeutic dose of DOX. These results, together with the adjuvant neoplastic effects of I_2_, lead us to propose Doxorubicin in combination with I_2_ supplement as a promising strategy against breast cancer progression.

## Methods

### Animals

The studies were performed on virgin female Sprague Dawley (200 g) rats from the vivarium of the Instituto de Neurobiología, UNAM-Juriquilla. Rats were housed in a temperature-controlled room (21 ± 1°C) with a 12-h/12-h light/dark schedule. Food (Purina rat chow; Ralston Purina Co., St. Lous, MO) and water were available ad libitum. All of the procedures followed UNAM and Use Committee Guidelines.

### Carcinogen, iodine, and DOX treatments

At 6 weeks of age, rats were treated to induce mammary tumors with a single ip injection of 50 mg/kg MNU (Sigma, St. Louis, MO; dissolved in 0.9% saline, pH 5.0 and activated by heating to 50-60°C) [40]. Rats were weighed and palpated for tumors every week beginning 1 month after MNU exposure. When the tumors grew to 2–3 cm^3^ (12–16 weeks later), the animals were divided into the following groups: Control, DOX16 (16 mg/kg), DOX8 (8 mg/kg), DOX4 (4 mg/kg); DOX16 + 0.05% I_2_ (DOX16-I_2_), DOX8 + 0.05% I_2_ (DOX8-I_2_), DOX4 + 0.05% I_2_ (DOX4-I_2_), and 0.05% I_2_ (I_2_). Each dose of DOX (Pharmacia & Upjohn) was administered in a single ip injection. The I_2_ stock solution is a saturated solution (1.33 mM) of iodine sublimate (J.T. Baker; Edo. de México, Mexico) in distilled water, and the concentration of 0.05% was confirmed by titration with sodium thiosulfate (40). The drinking water I_2_ solution was delivered in amber bottles that were changed every two days. After correction for sublimation (<30%), we estimated that animals consume an intake equivalent to that of Asian populations (5–7 mg/day). The I_2_ supplement in drinking water began 2 days before DOX and continued for another 5 days until the animals were sacrificed. To determine the long-term effect of DOX4-I_2_ treatment, a second series of experiments was carried out, with or without I_2_ being supplemented continuously for 56 days, and DOX4 was administered by ip injection on day 0 and/or on day 14. The treatment groups were designed as: single injection day 0 DOX4 (DOX4); I_2_ alone (I_2_); DOX4 + I_2_ (DOX4/I_2_); DOX4 + second injection at day 14 of DOX4 (DOX4 + DOX4); I_2_ with only the day 14 injection of DOX4 (I_2_ + DOX4), and DOX/I_2_ with the second injection of DOX4 on day 14 (DOX4/I_2_ + DOX4). A tumor was defined as a discrete palpable mass recorded for least two consecutive weeks. Tumor sizes were measured using calipers, and the volumes were calculated by the ellipsoid formula [41].

### Immunohistochemistry of proliferating cell nuclear antigen (PCNA)

Five-μm sections of mammary tumors from rats with or without DOX16 or I_2_ treatment were deparaffinized, rehydrated, and subjected to antigen retrieval (10 mM sodium citrate) at 80°C for 20 min. After cooling at room temperature, sections were treated with 0.3% hydrogen peroxide to block endogenous peroxidase activity. Non-specific binding was blocked with 2% non-fat dry milk in 20% fetal bovine serum-PBS solution (1 hr at 37°C). Sections were incubated at room temperature for 30 min in a humid chamber with mouse monoclonal anti-rat PCNA, clone PC10 (DakoCytomation, Carpinteria, CA), diluted 1:150). Immune complexes were visualized by goat anti-mouse-immunoglobulin, peroxidase labeled (EnVision™ + System, peroxidase, DakoCytomation, Carpinteria, CA). Diaminobenzidine (DAB) was used as the chromogen to generate a brown precipitate after reaction with peroxidase. Sections were counterstained with hematoxylin, rinsed, dehydrated, and mounted with Entellan (Merck, Darmstadt, Germany). Tumor sections were incubated without either the primary or secondary antibody to test for antibody specificity. A brown stain over the nucleus identified PCNA-positive cells. Labeling indices were obtained by counting the number of labeled cells among at least 500 cells per region, and 5 randomly selected regions were analyzed.

### Real time PCR

PPARγ, Bax, Bcl2, and Surv expression were analyzed by quantitative real time PCR (qPCR) from tumors after short- (7 day) or long- (56 day) term treatment. Total RNA was obtained using the TRIzol reagent (Life Technologies, Inc., Carlsbad, CA) dissolved in RNAase-free water (50 μL), and stored at -70°C. The extracted RNA (2 μg) was reverse transcribed using oligo-deoxythymidine. In order to eliminate genomic DNA contamination, we carried out the RT assay for each individual sample, and as control we used one tube that contained an aliquot from a pool of all samples but no transcriptase enzyme (-RT). We ran a standard PCR for each pair of oligos with two individual samples (random) and the -RT control. The sequence detector system Roto-Gene 3000 (Corbett Research, Mortlake, NSW, Australia) was used to perform qPCR with SYBRgreen as a marker for DNA amplification. The reaction was carried out with 1 μL of cDNA template and the qPCR supermix-UDG kit (Invitrogen), using 40 cycles of three-step amplification (94°C for 30 s, 55–60°C for 30 s, 72°C for 30 s) and the gene-specific primers listed in Table 1. PCR generated only the expected specific amplicon, which was demonstrated in each case by the melting temperature profile (dissociation curve) and by electrophoresis of 5 μL of the PCR product through a 2% agarose gel containing ethidium bromide in TAE buffer. No PCR products were observed in the absence of template. Gene expression was calculated using the D-cycle threshold (Dct) method and normalized to the content of β-actin, a non-regulated housekeeping gene [42]. The coefficient of variation for this gene was less than 15% in all RT-PCR assays, indicating that the significant changes observed in the different groups correspond to changes in the experimental genes.

**Table 1:** Oligonucleotides

**Gen**	**Reference**	**Sense**	**Antisense**	**pb**
PPAR-γ	AF156665	tcaaaccctttaccacggtt	caggctctactttgatcgca	147
β-actin	NM031144	gtcccagtatgcctctggtcgtac	ccacgctcggtcaggatcttcatg	171
Bax	NM017059	cagggaggatggctgggaga	ccagacaagcagccgctcacg	352
Bcl-2	NM016993	cgaagtgctattggtacctg	tatttgtttggggcaggtct	780
Surv	NM022274	aagccacttgtcccagctt	ctcatccactcccttcctc	198

### Creatine kinase MB (CK-MB) and catalase activity

Serum levels of CK-MB were assayed to evaluate cardiac damage, using the commercial kit Humazym M-Test (Human GmbH-65205 Wiesbaden, Germany). Cardiac catalase activity was assayed using the method described by Aebi [43]. Heart tissue was homogenized in assay buffer (50 mM KH_2_PO_4_, pH 7), and the homogenates were centrifuged at 6000 × g at 4°C for 20 min. The supernatant was diluted with 3 volumes of the assay buffer. Specific activity is expressed as μmol H_2_O_2_/min/mg protein. Protein was determined using the Bradford method (Bio-Rad protein assay; Hercules, CA) with bovine serum albumin (BSA) as standard.

### Cardiac lipoperoxidation

The concentrations of metabolites related to lipoperoxidation were quantified in heart tissue by the thiobarbituric acid reaction and expressed as nanomoles of malondialdehyde (MDA) per mg protein [44]. All manipulations were made rapidly on ice to avoid nonspecific peroxidation. Some modifications to the original method were introduced. To determine basal levels, a sample of homogenate (0.5-1 mg protein) was incubated for 30 min at 37°C in a 1-mL volume containing 150 mM Tris buffer, pH 7.4; incubation was stopped by adding 1.5 mL 20% acetic acid (adjusted to pH 3.5 with KOH) and 1.5 mL 0.8% thiobarbituric acid. Samples were kept for 45 min in a boiling water bath, and 1 mL 2% KCl was added to each sample at the end of the incubation. The colored complex formed was extracted with butanol-pyrimidine (1:1, v/v) and detected at 532 nm. Protein was quantified by the Bradford method as above.

### Iodine antioxidant effect

Iodine antioxidant power was measured *in vitro* using the ferric reducing/antioxidant power assay (FRAP) method reported by Benzie and Strain [45] with some modifications. The FRAP reagent was freshly prepared prior to each analysis by combining 300 mM sodium acetate buffer pH 3.6, 10 mM 2,4,6-tripyridyl-s-triazine in 40 mM HCl, and 20 mM ferric chloride in the proportions 10:1:1 (v/v/v). A series of ascorbic acid standards was freshly prepared for each analysis. The FRAP assay was carried out in a microplate (Dynex Technologies, Inc) Individual wells were manually loaded with sodium acetate buffer (60 μL) and either ascorbic acid standards or samples (60 μL), after which 240 μL FRAP reagent was added to each well; absorbance at 620 nm was determined 4 min later. Different chemical forms of iodine were analyzed and compared to ascorbic acid as standard.

### Statistical analysis

The data are expressed as mean ± SD. Differences between experimental groups were analyzed using a one-way ANOVA and Tukey’s honest significant difference test. Differences with p < 0.05 were considered statistically significant.

### Histological heart microscopy

Heart fragments were fixed at 4°C by immersion in fixative solution containing 3% glutaraldehyde either in cacodylate or phosphate buffer at pH 7.2 and processed by routine electron microscopy techniques [46]. Epon 1-μm thick sections were obtained with a glass knife and thin sections with a diamond knife either in a Reichert Um03 or RMC ultramicrotome. Thin sections were contrasted with uranyl acetate and lead citrate. One-μm sections were stained with 1% toluidine blue in 0.1 M sodium borate solution at alkaline pH. Thin sections were observed in a JEOL 1010 electron microscope operated at 80 KV.

## Abbreviations

MC: Mammary cancer; I2: Molecular iodine; Dox: Doxorubicin; MNU: Methylnitrosourea; PPARγ: Peroxisome proliferator-activated receptor type gamma; PCNA: Proliferating cell nuclear antigen; 6-IL: 6-iodolactone; Surv: Survivin; CK-MB: Creatine kinase MB; ROS: Reactive oxygen species; MDA: Malondialdehyde; qPCR: Quantitative real time polymerase chain reaction; FRAP: Ferric reducing/antioxidant power assay; LPO: Lipoperoxidation.

## Competing interests

The authors declare that they have no competing interests.

## Authors’ contributions

YA carried out the tumorogenesis induction and animal experiments and prepared the manuscript. GD performed the real-time polymerase chain reaction analysis. AC performed the microscopic analysis of heart. BA participated in the design of the study and performed the statistical analysis, and CA participated in the study design and coordination. All authors read and approved the final manuscript.

## Supplementary Material

Additional file 1 **Cardiac electron micrography. **Animals with tumors (2–3 cm^3^) received a single injection of DOX16 and/or 0.05% I_2 _treatment (drinking water) for 7 days. A, mitochondrion and fiber damage; B, laminary rearrangement. Click here for file
