# Supplementary figures and images for: Iodine and doxorubicin, a good combination for mammary cancer treatment: antineoplastic adjuvancy, chemoresistance inhibition, and cardioprotection
Source: Mol Cancer. 2013 May 24;12:45. doi: 10.1186/1476-4598-12-45 (PMC3673826; doi:10.1186/1476-4598-12-45)

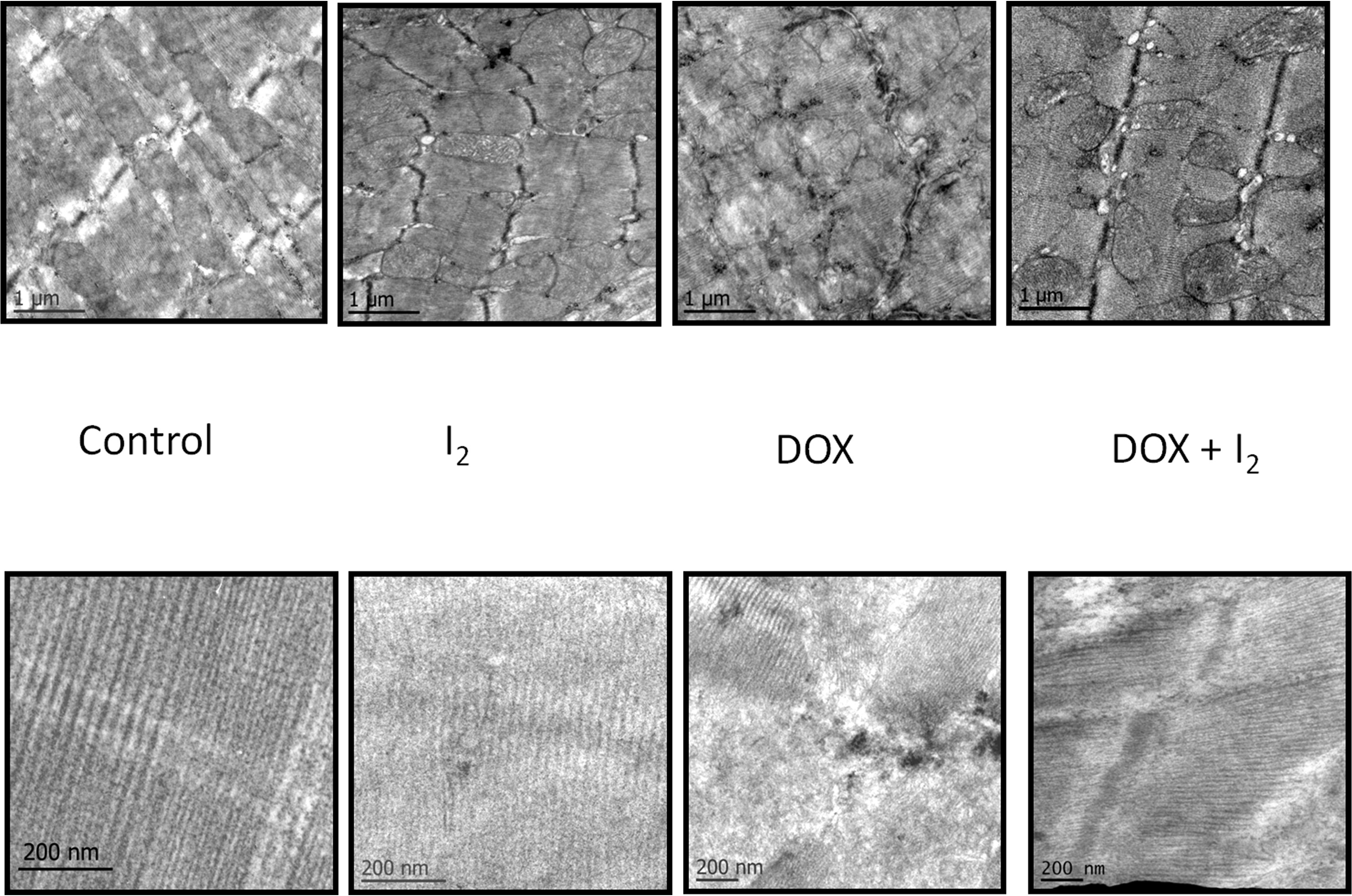

Supplement: Additional file 1 — Cardiac electron micrography. Animals with tumors (2–3 cm3) received a single injection of DOX16 and/or 0.05% I2 treatment (drinking water) for 7 days. A, mitochondrion and fiber damage; B, laminary rearrangement. [file 1476-4598-12-45-S1.jpeg]
